# Longitudinal effects of emerging adults’ self-presentation on social networking sites on fear of missing out

**DOI:** 10.1186/s40359-026-04358-z

**Published:** 2026-03-12

**Authors:** Shuqing Wang, Yuru Jing, Qiqi Yu, Fengxia Yan, Bijuan Huang

**Affiliations:** 1 School of Education and Psychology, University of Jinan, No. 336 Nanxinzhuang West Road, Jinan City, Shandong Province People’s Republic Of China; 2 Student Affairs Department, Student Mental Health Education Center, Shandong Jianzhu University, Jinan, Shandong People’s Republic of China

**Keywords:** Fear of missing out, Self-presentation on social networking sites, Online social comparison, Basic psychological needs satisfaction, Neuroticism

## Abstract

**Background:**

The widespread use of social networking sites may increase fear of missing out among emerging adults. However, few longitudinal studies have examined how self-presentation on social networking sites influences fear of missing out or the underlying mechanisms from an individual-context interaction perspective. This study examined the longitudinal mediating roles of online social comparison and basic psychological needs satisfaction in the relationship between self-presentation on social networking sites and fear of missing out, as well as the moderating effect of neuroticism.

**Methods:**

A three-wave longitudinal design was conducted with 743 Chinese college students (*M*_age_ = 19.57) recruited through cluster sampling. Participants completed self-report questionnaires assessing self-presentation on social networking sites, online social comparison, basic psychological needs satisfaction, neuroticism and fear of missing out. Data were analyzed using serial mediation modeling and moderated mediation modeling.

**Results:**

Positive and authentic self-presentation on social networking sites exerted differential effects on fear of missing out through distinct pathways. Specifically, positive self-presentation showed dual effects on fear of missing out, reducing fear of missing out via basic psychological needs satisfaction while simultaneously increasing fear of missing out through a serial pathway involving online upward social comparison and reduced basic psychological needs satisfaction. This serial indirect effect was stronger among individuals with higher levels of neuroticism. In contrast, authentic self-presentation reduced fear of missing out only through basic psychological needs satisfaction.

**Conclusions:**

Positive self-presentation on social networking sites shapes fear of missing out through both protective and risk pathways, with basic psychological needs satisfaction functioning as a protective factor and online upward social comparison operating as a risk mechanism that is amplified among individuals with higher levels of neuroticism. In contrast, authentic self-presentation on social networking sites reduces fear of missing out primarily through enhanced basic psychological needs satisfaction.

## Introduction

A prevalent negative emotional experience among emerging adults has been identified, termed the fear of missing out (FoMO) [1]. FoMO is a pervasive anxiety that arises from concerns about missing others’ novel experiences or positive events [2]. According to a survey by the China Internet Network Information Center, as of December 2024, the average weekly internet usage per person reached 28.7 hours [3], indicating that social media has deeply integrated into their lives. This may heighten the feeling of FoMO which reflects the adverse psychological impact of social networking sites (SNSs) use on individuals’ affect and self-perception. Individuals with higher levels of FoMO have been shown to exhibit reduced self-evaluation and diminished self-esteem, as well as a greater risk of problematic social media use and internet addiction [1, 4].

Based on the ecological techno-subsystem theory, the online environment has become an important context shaping individuals’ psychological development [5]. Empirical studies have consistently shown that intensive SNSs engagement exacerbates FoMO [6–10]. As a primary mode of SNSs interaction, self-presentation on SNSs refers to a series of behaviors through which individuals strategically manage their self-image to influence others’ perceptions. Based on information disclosure strategies, self-presentation on SNSs is commonly categorized into positive self-presentation (selectively presenting positive information about oneself) and authentic self-presentation (presenting only the most authentic self without choosing content) [11, 12]. Drawing on the uses and gratifications theory, individuals use SNSs primarily to satisfy specific psychological needs [13]. Individuals present idealized selves on SNSs as a strategy to fulfill psychological needs that are insufficiently met in offline contexts [14]. This compensatory mechanism subsequently increases the frequency of positive self-presentation behaviors, while simultaneously fostering excessive concern with others’ feedback and evaluations. Such behavioral patterns involve heightened sensitivity to self-relevant social cues. Over time, they may exacerbate FoMO [15], as frequent exposure to social media increases upward social comparison and anxiety about social evaluation [16].

Nevertheless, positive self-presentation is not inherently maladaptive. When it elicits supportive responses from others, it may enhance self-esteem and promote psychological well-being [17], suggesting that its effects on FoMO may depend on contextual and interpersonal conditions. By contrast, authentic self-presentation allows individuals to share their true selves and obtain positive social feedback, which may facilitate social support [18] and, in turn, mitigate FoMO [19, 20]. Taken together, positive and authentic self-presentation on SNSs may exert differential effects on FoMO. Their effects depend on contextual features and individual differences [16, 21]. This highlights the need to clarify whether the two forms of self-presentation operate through different psychological mechanisms. Despite these insights, little empirical research has examined how self-presentation on SNSs relates to FoMO. In particular, it remains unclear whether positive and authentic self-presentation have different effects and whether these effects operate through distinct psychological mechanisms.

In order to address this gap, the present study employs a longitudinal design to systematically examine the distinct impacts of positive and authentic self-presentation on SNSs on FoMO, and to investigate their respective internal mechanisms. Developmental contextualism emphasizes the dynamic interaction between individuals and their environment. Guided by this perspective, this study proposes a model of how FoMO is influenced by the interplay between online social networking environments and individual factors. Specifically, self-presentation on SNSs may affect FoMO through three pathways: cognitive processes (e.g., online social comparison), motivational mechanisms (e.g., basic psychological needs satisfaction), and personality traits (e.g., neuroticism) [22]. Accordingly, the present study investigates the impact of self-presentation on SNSs on FoMO, with particular attention to the roles of online social comparison, basic psychological needs satisfaction, and neuroticism. By identifying both protective and risk factors, this research aims to provide theoretical guidance for educational interventions targeting FoMO among emerging adults.

### The relationship among self-presentation on social networking sites, online social comparison, and fear of missing out

Social comparison has been identified as a critical mechanism through which SNSs use influences individuals’ emotional well-being, including subjective happiness, anxiety, and depression [16, 23]. Online social comparison can be defined as the process whereby individuals evaluate their own abilities and opinions by comparing themselves with others on digital platforms. In accordance with the principles of social comparison theory, the phenomenon may be categorized as follows: online upward social comparison, whereby individuals compare themselves with those regarded as superior; and online downward social comparison, whereby individuals compare themselves with those regarded as inferior [24]. Recent research has indicated that individuals’ self-presentation on SNSs, encompassing the dissemination of self-relevant information through various forms such as photos, texts, and videos, has the potential to amplify their propensity for social comparison [25]. When individuals engage in self-presentation on SNSs, they gauge their popularity and social competence through feedback mechanisms such as likes and comments. These mechanisms subsequently lead to both conscious and unconscious comparisons between an individual’s own posts and those of others [25–27]. It has been demonstrated that self-presentation behaviors on SNSs elicit stronger emotional involvement than passive browsing of others’ content, making individuals more attentive to peer self-representations and consequently more prone to social comparison behaviors [28, 29]. These upward or downward social comparisons subsequently trigger positive emotions (e.g., self-enhancement) or negative affective experiences (e.g., anxiety) [30–32]. According to the contrast effect in social comparison theory, upward social comparison can lead individuals to form negative self-evaluations and feel inferior. This process may then trigger feelings of frustration and worthlessness [33, 34]. This process has been shown to engender adverse emotional outcomes, including anxiety and depression, while concurrently exacerbating FoMO [28, 29, 33]. In contrast, the process of downward social comparison has been shown to facilitate psychological superiority through comparisons with less advantaged others [32, 34]. This process has been found to result in positive affect, enhanced self-esteem, and greater self-worth. This comparative mechanism has been demonstrated to serve as an effective buffer against stress and anxiety [35]. Therefore, the present study hypothesizes that online social comparison may mediate the relationship between emerging adults’ self-presentation on SNSs and FoMO.

However, it should be noted that the mechanisms behind the two forms of self-presentation on SNSs may differ. In the pursuit of social approval and positive feedback, individuals often resort to the presentation of “embellished” personal information. Such positive self-presentation on SNSs, when coupled with social feedback (e.g., comments, likes), facilitates upward social comparison, thereby exacerbating FoMO [36, 37]. In contrast to positive self-presentation, authentic self-presentation on SNSs has been demonstrated to enhance self-esteem and to reduce FoMO by decreasing problematic SNSs use [38–40]. These studies suggest that authentic self-presentation may result in distinct patterns of online social comparison. However, empirical research specifically examining the mediating role of online social comparison in the relationship between self-presentation on SNSs and FoMO is still insufficient and requires further investigation.

### The relationship among self-presentation on social networking sites, basic psychological needs satisfaction and fear of missing out

Basic psychological needs satisfaction (BPNS), as derived from self-determination theory, posits that human well-being is contingent on the fulfillment of three innate psychological needs: competence, autonomy, and relatedness [41]. When the social environment satisfies an individual’s psychological needs, the individual is able to actively engage and adapt to new environments, thereby maintaining physical and mental health [42, 43]. Conversely, when such needs remain unfulfilled, individuals may encounter adverse emotional states, including depression and anxiety [43, 44].

According to the uses and gratifications theory, individuals primarily engage with SNSs to fulfil specific psychological needs [13]. Within this theoretical framework, self-presentation on SNSs serves as a key way for satisfying users’ basic psychological needs [45, 46]. Specifically, SNSs enable users to freely select shared content and self-presentation styles (e.g., showcasing positive attributes or making authentic self-disclosures), thereby satisfying their need for autonomy [47, 48]. Furthermore, self-presentation on SNSs can enhance friendship quality, facilitate social recognition and support, and foster feelings of security and belonging. This satisfies individuals’ relatedness needs. Consequently, self-presentation on SNSs contributes significantly to emerging adults’ BPNS [40, 49].

Extensive research has demonstrated that BPNS negatively predicts individuals’ FoMO levels [2, 50]. According to self-determination theory, effective self-regulation depends on the satisfaction of three basic psychological needs: autonomy, competence, and relatedness [51]. From this self-regulation perspective, Przybylski et al. regard FoMO as a manifestation of disrupted self-regulation [2]. Similarly, Chai et al. argue that when an individual’s basic psychological needs are not fulfilled, his or her self-regulatory capacity will be impaired, thereby leading to FoMO [52]. Previous studies have mainly examined pairwise relationships between SNS self-presentation, BPNS, and FoMO. However, few studies have explored how all three constructs are interrelated. Drawing on self-determination theory, BPNS is therefore proposed as a potential mediating mechanism linking self-presentation on SNSs to FoMO.

### The serial mediation effects of online social comparison and basic psychological needs satisfaction

Social comparison constitutes a critical factor influencing individuals’ BPNS [37, 53]. In the context of upward social comparison, individuals’ tendency to focus on the performance of superior others has been shown to evoke feelings of incompetence [54]. This phenomenon has been found to result in a decline in confidence and an exacerbation of feelings of inferiority. This directly impedes the satisfaction of competence needs and may ultimately trigger maladaptive behaviors such as aggression and hostility, thereby further undermining autonomy and relatedness needs [55–58]. In contrast, downward social comparison focuses on inferior others. This can protect or enhance the self-concept by highlighting discrepancies, generating positive affect, and ultimately promoting the satisfaction of basic psychological needs. Empirical evidence consistently demonstrates that downward social comparison more effectively supports needs satisfaction and fosters psychological well-being [37, 54]. The proliferation of SNSs has led to a significant escalation in the psychological impact of online social comparison. However, the question of whether these effects align with those observed in offline comparisons remains unresolved. This highlights the need to investigate how online upward and downward social comparisons relate to the BPNS, thereby extending the applicability of social comparison theory to digital contexts.

### The moderating role of neuroticism

Among core personality traits, neuroticism shows the strongest association with SNSs use [59, 60]. As Goldberg suggested, individuals with high levels of neuroticism tend to be more prone to negative affectivity and emotional instability [61]. This vulnerability to emotions such as fear and envy has been empirically supported by subsequent research [62]. Additionally, the study has found that neuroticism is a risk factor for FoMO and positively predicted its intensity [63].

Seidman discovers that the manner in which individuals present themselves on SNSs is dependent on their level of neuroticism [64]. Individuals with high neuroticism have been observed to post personal information (e.g., status updates) and emotional content (e.g., venting negative emotions) more frequently on SNSs. The cognitive-affective system theory of personality suggests that the same interpersonal scenario can trigger different cognitive and affective responses. These differences can lead to varied perceptions and behaviors across individuals [65]. Individuals with neurotic tendencies have been found to perceive online communication as more conducive than face-to-face interaction for obtaining social feedback and emotional comfort [66]. Consequently, they tend to engage in selective self-presentation behaviors when using SNSs [67]. Fox and Moreland’s seminal study shows that using SNSs such as Facebook leads people to engage in frequent social comparisons. These comparisons can ultimately trigger feelings of envy [68]. Furthermore, empirical studies have demonstrated that online social comparison tendencies are significantly influenced by neuroticism. Individuals with higher levels of neuroticism have been shown to exhibit stronger propensities for social comparison and greater susceptibility to envy [69, 70]. Moreover, research findings consistently indicate negative correlations between neuroticism and all three basic psychological needs [71]. Given the limited research in this area, the exact moderating role of neuroticism in the relationship between self-presentation on social networking sites, social comparison, BPNS and FoMO remains unclear. However, this represents an intriguing avenue for future research.

### The present study

Frequent SNSs use during emerging adulthood is strongly linked to the development of FoMO. This negative emotional experience can harm individuals’ mental health and well-being. Despite growing interest in FoMO, few empirical studies have systematically examined how self-presentation on SNSs influences FoMO. The psychological mechanisms behind this relationship also remain unclear. Importantly, different forms of self-presentation, especially positive and authentic self-presentation, may have distinct effects on FoMO. However, these effects are not yet fully understood.

Drawing on social comparison theory and self-determination theory, this study proposes two key psychological mechanisms linking self-presentation on SNSs to FoMO: online social comparison and BPNS. Moreover, individual differences in neuroticism may shape these processes by amplifying sensitivity to social comparison cues and need frustration, thereby moderating the indirect effects of self-presentation on FoMO.

To address these gaps, the present study adopts a three-wave longitudinal design over one year to examine (1) the differential effects of positive and authentic self-presentation on SNSs on FoMO, (2) the mediating roles of online social comparison and BPNS, and (3) the moderating role of neuroticism.

Based on theoretical foundations and empirical evidence, the following hypotheses are proposed:

- H1a: Positive self-presentation on SNSs is positively associated with FoMO through online social comparison.
- H1b: Authentic self-presentation on SNSs is negatively associated with FoMO through online social comparison.
- H2a: Positive self-presentation on SNSs is negatively associated with FoMO through increased BPNS.
- H2b: Authentic self-presentation on SNSs is negatively associated with FoMO through increased BPNS.
- H3a: Positive self-presentation on SNSs is indirectly associated with FoMO through a s serial mediating role of online social comparison and BPNS.
- H3b: Authentic self-presentation on SNSs is indirectly associated with FoMO through a serial mediating role of online social comparison and BPNS.
- H4a: Neuroticism moderates the associations between self-presentation on SNSs and online social comparison, such that these associations are stronger among individuals high in neuroticism.
- H4b: Neuroticism moderates the associations between online social comparison and BPNS, thereby conditionally influencing the indirect effects of self-presentation on SNSs on FoMO.

## Methods

### Participants

The present study employed a cluster sampling method to recruit emerging adults from three public universities in eastern China. Participants were instructed to undertake three surveys (T1: June 2021, *N* = 912; T2: December 2021, *N* = 857; T3: June 2022, *N* = 790). Due to leave of absence, invalid responses, and other reasons, a total of 743 participants (aged between 18 and 22 years, *M*_age_ = 19.57, *SD* = 1.20) were successfully tracked across all three waves of data collection. The sample comprised 185 male and 558 female emerging adults. Inherent differences in the gender composition across the surveyed universities and academic majors resulted in a substantial gender imbalance in the final sample. Based on existing theories and the design of the present study, self-presentation on SNSs was measured at Time 1 (T1), online social comparison, BPNS, and neuroticism at Time 2 (T2), and FoMO at Time 3 (T3).

There were no significant differences between attrited and retained participants on the key variables (*p*s > 0.17). The result of Little’s MCAR test was significant (*p* < 0.001), indicating that the missing data were not completely random. Although there were no significant differences in data completeness (0 = incomplete, 1 = complete) for age (*t* = − 0.52, *p* = 0.46), T1 positive self-presentation on SNSs (*t* = − 0.40, *p* = 0.69), T1 authentic self-presentation on SNSs (*t* = 0.10, *p* = 0.92 ), T2 online upward social comparison (*t* = − 0.23, *p* = 0.82), T2 online downward social comparison (*t* = − 0.16, *p* = 0.87), T2 BPNS ( *t* = 0.43, *p* = 0.66), T2 neuroticism(*t* = − 0.14, *p* = 0.89), and T3 FoMO (*t* = − 0.43, *p* = 0.66), a significant difference was observed for gender (*χ*^2^*/df* = 4.13, *p* = 0.03). Compared to complete data, the proportion of female students was higher in incomplete data. Therefore, the present missing pattern was likely to be MAR. Multiple imputation methods were employed to handle missing data.

### Measures

#### Fear of missing out

FoMO was measured using the Fear of Missing Out scale of Przybylski et al. [2], which was revised by Zhang et al. [72]. The scale consists of 10 items, such as, “I worry that other people have more exciting experiences than I do”. Each item is rated on a 5-point Likert scale ranging from “strongly disagree” to “strongly agree”, with higher total scores indicating higher levels of FoMO. Previous studies had demonstrated the scale had high reliability and validity among Chinese adolescent groups [72]. In the present study, confirmatory factor analysis (CFA) showed that the scale at T3 had good construct validity ( *χ*^2^*/df* = 4.71, CFI = 0.96, TLI = 0.93, RMSEA = 0.07, SRMR = 0.05). The Cronbach’s α coefficient for T3 FoMO was 0.84.

#### Self-presentation on social networking sites

Self-presentation on SNSs was measured using the Authentic and Positive Self-Presentation Scale of Kim and Lee [12], which was revised by Niu et al. [73]. The scale consists of 10 items, including positive self-presentation on SNSs (four items, e.g., “I only post pictures that show me at my happiest”) and authentic self-presentation on SNSs (six items, e.g., “I don’t mind posting photos of myself when I don’t look happy”). It is rated on a 7-point Likert scale ranging from “strongly disagree” to “strongly agree”, with higher scores representing higher levels of authentic or positive self-presentation on SNSs. Previous studies had demonstrated that CFA results of the Chinese version of the scale showed a good fit [73]. In the present study, CFA showed that the scale at T1 had good construct validity (*χ*^2^*/df* = 4.95, CFI = 0.95, TLI = 0.92, RMSEA = 0.07, SRMR = 0.05). The Cronbach’s α coefficients for T1 positive and authentic self-presentation on SNSs were 0.82 and 0.77, respectively.

#### Online social comparison

Online social comparison was measured using the social comparison subscale from the Iowa-Netherlands Comparison Orientation Measure, which was revised by Bai et al. [74, 75]. Each item was modified to reflect social comparisons in the context of SNSs, such as “On social networking sites, I often like to compare myself with those who are doing better than me” and “On social networking sites, I often like to compare myself with those who are doing worse than me”. The scale comprises two distinct dimensions: online social upward comparison and online social downward comparison. Each dimension is measured by six items. The items are scored on a 5-point scale ranging from “strongly disagree” to “strongly agree”, with higher scores denoting a higher level of online upward and downward social comparison. The revised scale had good construct validity among Chinese adolescent populations [75]. In the present study, CFA showed that the scale at T2 had good construct validity ( *χ*^2^*/df* = 3.22, CFI = 0.98, TLI = 0.98, RMSEA = 0.06, SRMR = 0.02). The Cronbach’s α coefficients for T2 online upward and downward social comparison were 0.90 and 0.94, respectively.

#### Basic psychological needs satisfaction

The Chinese version of the Basic Psychological Needs Questionnaire, revised by Yu (2012) based on Deci and Ryan’s original version [41, 76]. It includes three dimensions: relatedness needs, competence needs, and autonomy needs, with a total of 21 items. The scale is rated on a 7-point scale ranging from “strongly disagree” to “strongly agree”. Higher scores indicate a greater degree of satisfaction of the three basic psychological needs. Previous studies had demonstrated CFA results indicated that the structural model of the scale fitted well [73]. CFA in the present study showed that the scale at T2 had good construct validity ( *χ*^2^*/df* = 4.91, CFI = 0.93, TLI = 0.90, RMSEA = 0.07, SRMR = 0.07). The Cronbach’s α coefficients for the three dimensions and total scale at T2 were 0.68, 0.72, 0.80 and 0.87 respectively.

#### Neuroticism

The Chinese version of the Neuroticism subscale from the Big Five Inventory-2, revised by Zhang et al. based on the original version by Soto & John, was utilized [77, 78]. The subscale is comprised of 12 items, which are scored on a 5-point scale ranging from “strongly disagree” to “strongly agree”. Higher scores indicate a higher level of neuroticism. In a sample of Chinese university students, the Chinese version of BFI-2 demonstrated good reliability and validity [78]. In the present study, CFA showed that the scale at T2 had good construct validity ( *χ*^2^*/df* = 5.59, CFI = 0.93, TLI = 0.90, RMSEA = 0.07, SRMR = 0.05). The Cronbach’s α coefficient for T2 neuroticism was 0.87.

### Procedure

The study was approved by the Ethics Committee of School of Education and Psychology, University of Jinan (NO. 201709003). To ensure the quality of data collection, all experimenters received comprehensive training covering standardized administration procedures. The training included learning the standardized instructions used to introduce the study’s purpose, confidentiality principles, and participants’ rights to voluntary participation and withdrawal. Experimenters were also trained to ensure that each participant fully understood and signed the informed consent form, to administer questionnaires in the prescribed order to prevent omissions or errors. These measures were implemented to minimize extraneous variables arising from inconsistencies in procedural operations.

The study adopted an on-site group paper-and-pencil testing procedure. Each session was conducted by two or three experimenters working collaboratively, with one responsible for administering the procedure and the other for monitoring the testing environment. The administration strictly followed standardized procedures: obtaining informed consent, delivering uniform instructions, distributing questionnaires in a fixed order while controlling for time, ensuring independent completion, and collecting and checking the questionnaires immediately after completion. Testing session lasted approximately 10–20 min. All procedures adhered to ethical guidelines. Participants who successfully completed the test were presented with a $0.5 ballpoint pen gift.

### Data analysis

All data were collected and analyzed using SPSS 22.0 and Mplus 8.4 [79, 80].

Firstly, descriptive statistics, correlation analysis and so on were performed in SPSS 22.0.

Subsequently, Mplus 8.4 was used to investigate the link between self-presentation on SNSs and FoMO. A structural equation model (SEM) was employed to evaluate the path including T1 self-presentation on SNSs, T2 online social comparison, T2 BPNS, and T3 FoMO. The model fit index included: the Comparative Fit Index (CFI), the Tucker-Lewis index (TLI), the Root Mean Square Error of Approximation (RMSEA) and Chi-square. The model fit was regarded as acceptable if the CFI and TLI were above 0.90, and the RMSEA was less than 0.08 [81].

Then, the study proposed the construction of a moderated serial mediation model in order to examine the moderating effect of T2 neuroticism. Given the potential influence of gender on research outcomes, gender was incorporated as a control variable.

In the preceding analysis, to simplify the model structure and enhance both goodness-of-fit and estimation stability, with the exception of the BPNS, measured by three dimensions as observed indicators, all other variables were operationalized using item parceling through the balancing method to generate observed indicators [82].

## Results

### Preliminary analysis

The descriptive statistics and correlation analysis for all variables were presented in Table 1. Specifically, T1 positive self-presentation on SNSs displayed a significant positive correlation with T2 online upward social comparison and T3 FoMO, and significantly negatively correlated with T1 authentic self-presentation on SNSs. Moreover, a significant positive correlation was identified between T1 authentic self-presentation on SNSs and T2 BPNS. Additionally, T3 FoMO exhibited a substantial positive correlation with T2 online upward social comparison, T2 online downward social comparison and T2 neuroticism. Conversely, a significant negative correlation was observed with T2 BPNS. Furthermore, the correlations between the variables demonstrated that T2 neuroticism exhibited a positive correlation with both T2 online upward social comparison and T2 online downward social comparison. In addition, T2 neuroticism demonstrated a significant negative correlation with T1 authentic self-presentation on SNSs and T2 BPNS. Gender was significantly correlated with social downward comparison and BPNS.

**Table 1:** Descriptive statistics and correlation coefficients of study variables

Variables	M	SD	1	2	3	4	5	6	7	8
1. Age	19.57	1.20	1							
2. Gender	0.75	0.43	−0.12^**^	1						
3. T1 SNSs-P	3.97	1.14	−0.01	0.06	1					
4. T1 SNSs-A	4.33	1.16	−0.03	0.04	−0.37^**^	1				
5. T2 OSC-U	3.09	0.87	0.06	−0.01	0.12^**^	−0.01	1			
6. T2 OSC-D	2.08	0.74	0.05	−0.11^**^	0.02	−0.01	0.17^**^	1		
7. T2 BPNS	4.69	0.75	−0.03	0.15^**^	0.05	0.20^**^	−0.09^*^	−0.29^**^	1	
8. T3 FoMO	2.74	0.67	0.02	−0.05	0.16^**^	−0.07	0.23^**^	0.22^**^	−0.23^**^	1
9. T2 Neuroticism	2.89	0.65	−0.04	0.01	−0.02	−0.16^**^	0.21^**^	0.20^**^	−0.63^**^	0.28^**^

*SNSs* Social networking sites, *SNSs-P* Positive self-presentation on SNSs, *SNSs-A* Authentic self-presentation on SNSs, *OSC-D* Online downward social comparison, *OSC-U* Online upward social comparison, *BPNS* Basic psychological needs satisfaction, *FoMO* Fear of missing out

Gender was coded as male = 0, female = 1; ^*^ *p*<0.05, ^**^p<0.01

### Longitudinal mediation between self-presentation on social networking sites and fear of missing out

**Fig. 1:**
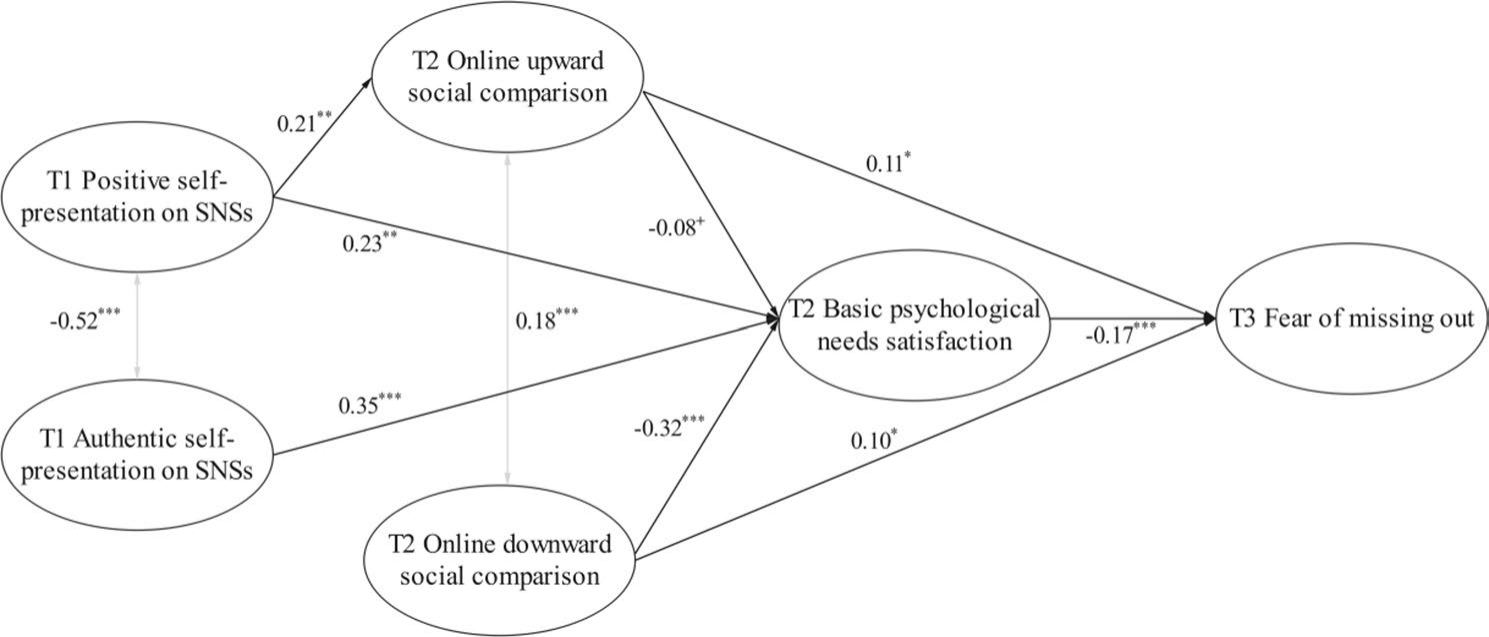
The serial mediation model of the impact of self-presentation on social networking sites on fear of missing out. Notes. ^*^ *p*<0.05, ^**^ *p* < 0.01, ^***^ *p* < 0.001, ^+^ 0.05 < *p* < 0.1. To maintain the simplicity of the model, non-significant paths are not depicted

The serial mediation model was constructed to examine the roles of T2 online social comparison and BPNS in the effects of T1 positive and authentic self-presentation on SNSs on T3 FoMO. The mediation effect was tested using the bias-corrected bootstrap method. This model exhibited satisfactory model fit indices (*χ*^2^*/df* = 3.01, CFI = 0.97, TLI = 0.96, RMSEA = 0.05, SRMR = 0.07). The results indicated that, after controlling for T1 FoMO, T1 positive self-presentation on SNSs significantly predicted T2 online upward social comparison (*β* = 0.21, *p* < 0.01); T1 positive and authentic self-presentation on SNSs significantly predicted T2 BPNS (*β*_SNSs−P_ = 0.23, *β*_SNSs−A_ = 0.35, *p*s < 0.01). T2 online upward social comparison and T2 BPNS showed a marginally significant relationship (*β* = −0.08, *p* = 0.075), and T2 online downward social comparison significantly predicted T2 BPNS (*β* = −0.32, *p* < 0.001). T2 online upward and downward social comparison and T2 BPNS significantly predicted T3 FoMO (*β*_OSC−U_ = 0.11, *p* < 0.05; *β*_OSC−D_ = 0.10, *p* < 0.05; *β*_BPNS_ = − 0.17, *p* < 0.001). However, T1 positive and authentic self-presentation on SNSs did not significantly predict T2 online downward social comparison (*β*_SNSs−P_ = 0.06, *p* = 0.39; *β*_SNSs−A_ = 0.02, *p* = 0.71) or T3 FoMO (*β*_SNSs−P_ = 0.09, *p* = 0.18; *β*_SNSs−A_ = 0.034, *p* = 0.62), and T1 authentic self-presentation on SNSs did not significantly predict T2 online upward social comparison(*β* = 0.10, *p* = 0.18) (see Fig. 1 ).

Three mediating paths were revealed in the relationship between positive self-presentation on SNSs and FoMO (see Table 2 ): (1) the mediation effect of T1 positive self-presentation on SNSs → T2 online upward social comparison → T3 FoMO had a 95% bias-corrected bootstrap confidence interval excluding zero (*β* = 0.023, 95% CI = [0.004, 0.062]); (2) The mediation effect of T1 positive self-presentation on SNSs → T2 BPNS → T3 FoMO had a 95% bias-corrected bootstrap confidence interval excluding zero (*β* = −0.038, 95% CI = [− 0.083, − 0.012]). Results indicated that T2 online upward social comparison and T2 BPNS served significant mediating roles in the relationship between T1 positive self-presentation on SNSs and T3 FoMO. (3) The serial mediation effect of T1 positive self-presentation on SNSs → T2 online upward social comparison → T2 BPNS → T3 FoMO had a 95% bias-corrected bootstrap confidence interval excluding zero (*β* = 0.003, 95% CI = [0.000, 0.011]), indicating that the serial mediation effect was significant.

**Table 2:** Mediation effect analysis of the serial mediation model

Path	β	Bootstrap 95%CI	
		LLCI	ULCI
T1 SNSs-P → T2 OSC-U → T3 FoMO	0.023	0.004	0.062
T1 SNSs-P → T2 OSC-D → T3 FoMO	0.008	−0.003	0.033
T1 SNSs-P → T2 BPNS → T3 FoMO	−0.038	−0.083	−0.012
T1 SNSs-P → T2 OSC-U → T2 BPNS → T3 FoMO	0.003	0.000	0.011
T1 SNSs-P → T2 OSC-D → T2 BPNS → T3 FoMO	0.004	−0.002	0.006
T1 SNSs-P → T3 FoMO Total indirect	0.001	−0.050	0.055
T1 SNSs-A → T2 OSC-U → T3 FoMO	0.011	−0.002	0.041
T1 SNSs-A → T2 OSC-D → T3 FoMO	0.005	−0.007	0.026
T1 SNSs-A → T2 BPNS → T3 FoMO	−0.059	−0.113	−0.023
T1 SNSs-A → T2 OSC-U → T2 BPNS → T3 FoMO	0.001	0.000	0.008
T1 SNSs-A → T2 OSC-D → T2 BPNS → T3 FoMO	0.003	−0.004	0.013
T1 SNSs-A → T3 FoMO Total indirect	−0.038	−0.100	0.013

*SNSs* Social networking sites, *SNSs-P* Positive self-presentation on SNSs, *SNSs-A* Authentic self-presentation on SNSs, *OSC-D* Online downward social comparison, *OSC-U* Online upward social comparison, *BPNS* Basic psychological needs satisfaction, *FoMO* Fear of missing out

In contrast to positive self-presentation, authentic self-presentation on SNSs predicted FoMO only indirectly through the mediating effect of BPNS (see Table 2). Specifically, the mediation effect of T1 authentic self-presentation on SNSs → T2 BPNS → T3 FoMO had a 95% bias-corrected bootstrap confidence interval excluding zero (*β* = −0.059, 95% CI = [− 0.113, − 0.023]), indicating that T2 BPNS served a significant mediating role in the relationship between T1 authentic self-presentation on SNSs and T3 FoMO.

### The moderating role of neuroticism

While controlling for T1 FoMO, the present study further examined the moderating role of T2 neuroticism in the serial mediation model involving T1 positive self-presentation on SNSs, T2 online upward social comparison, T2 BPNS, and T3 FoMO.

**Fig. 2:**
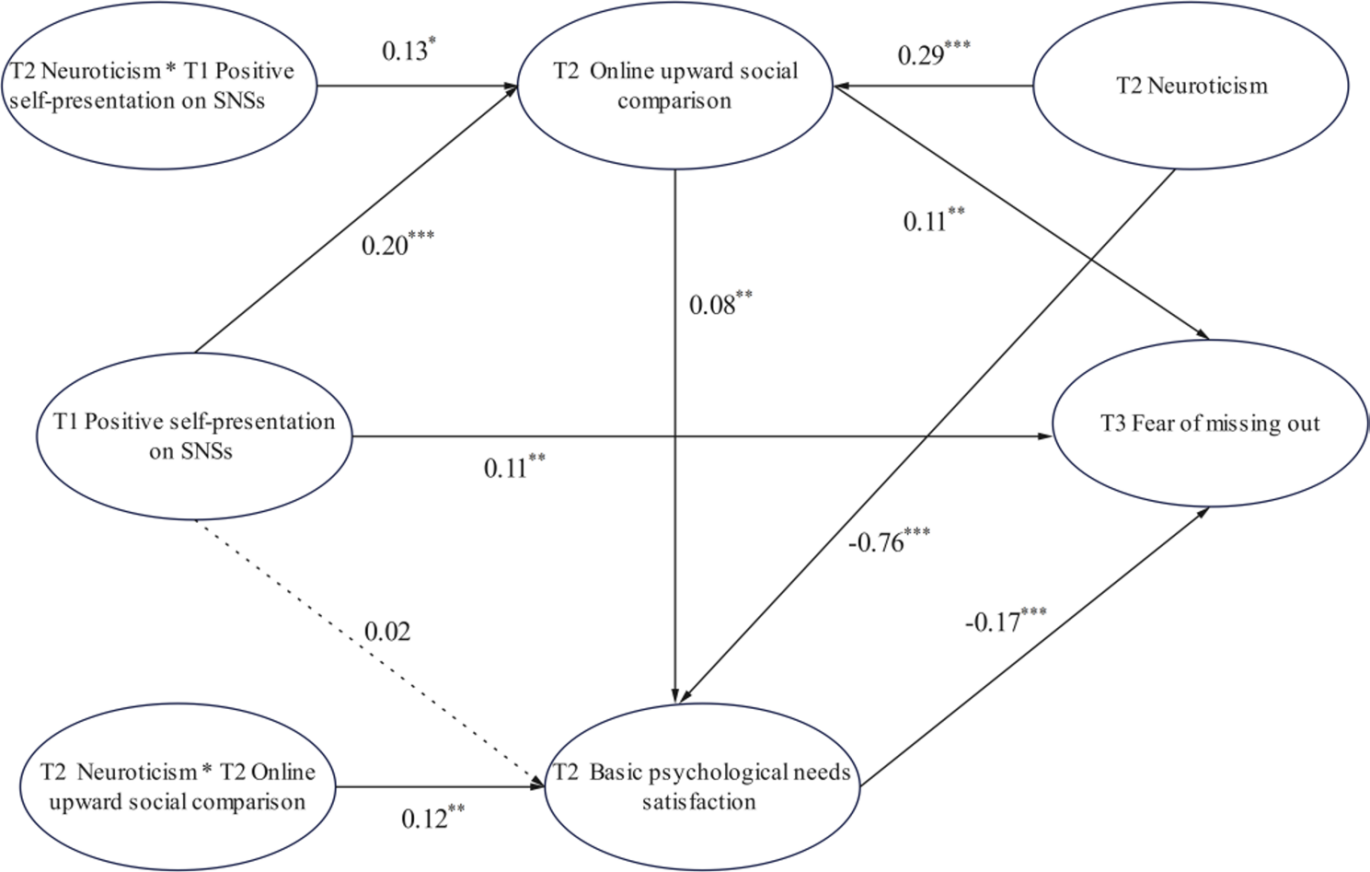
Moderated mediation model. Notes. ^*^ *p*<0.05, ^**^ *p* < 0.01, ^***^p < 0.001

In the model examining the effect of T1 positive self-presentation on SNSs on T3 FoMO, the mediating role of T2 online upward social comparison between T1 positive self-presentation on SNSs and T3 FoMO was statistically significant. Specifically, the first half of the path was *β* = 0.20, 95% CI [0.09, 0.31], while the second half of the path was *β* = 0.11, 95% CI [0.04, 0.19]. The mediation effect of T2 BPNS on the relationship between T1 positive self-presentation on SNSs and T3 FoMO was not significant (the first half path: *β* = 0.02, 95% CI [− 0.07, 0.10]; the second half path: *β* = −0.17, 95% CI [− 0.25, − 0.09]). The serial mediation effect of T2 online upward social comparison and T2 BPNS on the relationship between T1 positive self-presentation on SNSs and T3 FoMO was significant (indirect path: *β* = 0.08, 95% CI [0.02, 0.14]). The interaction term between T2 neuroticism and T1 positive self-presentation on SNSs significantly predicted T2 online upward social comparison (*β* = 0.13, 95% CI [0.01, 0.24]). The interaction term between T2 neuroticism and T2 online upward social comparison significantly predicted T2 BPNS (*β* = 0.16, 95% CI [0.05, 0.18]), indicating that T2 neuroticism moderate the relationship between T1 positive self-presentation on SNSs and T2 online upward social comparison, as well as the relationship between T2 online upward social comparison and T2 BPNS (see Fig. 2).

**Fig. 3:**
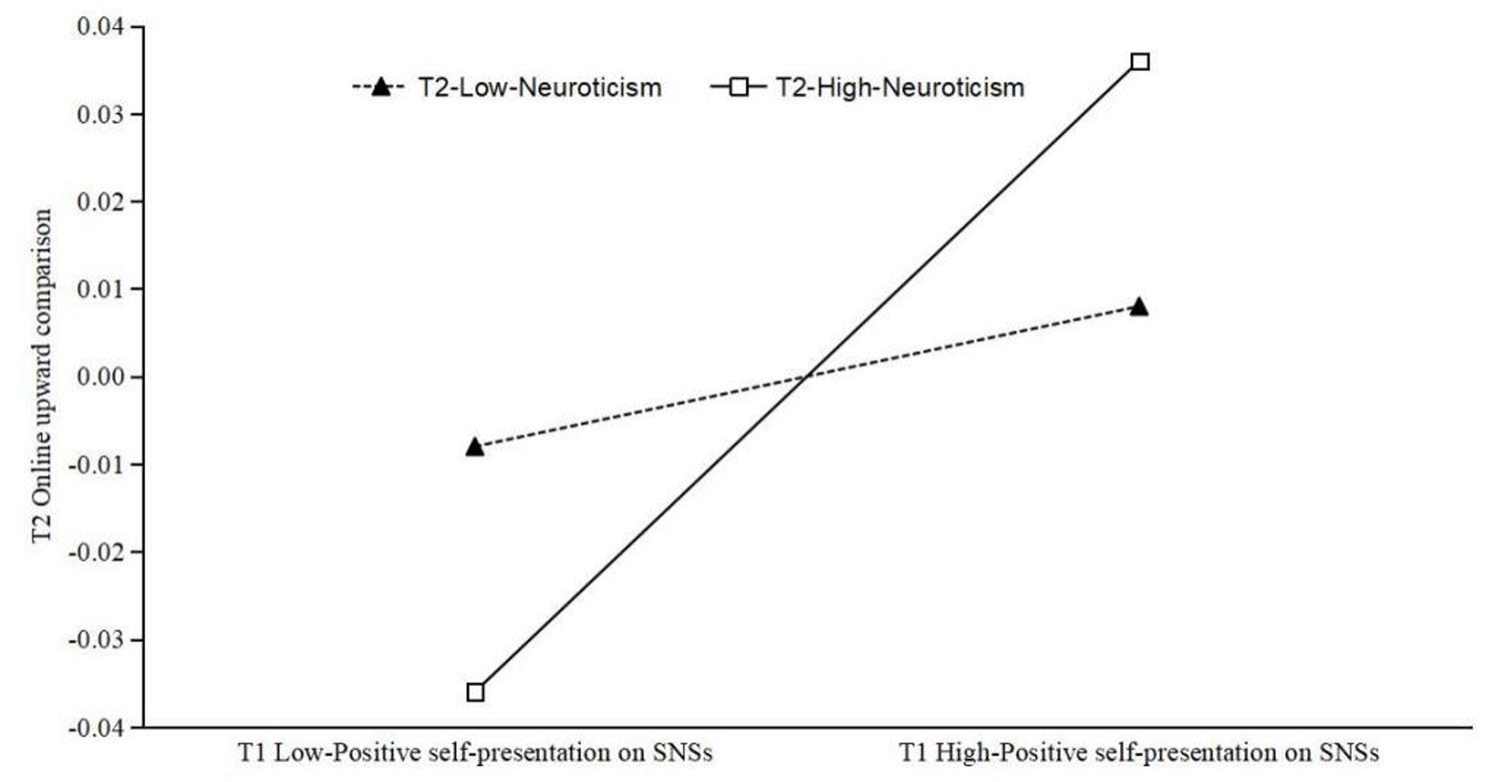
Simple slope analysis

The moderating effect of T2 neuroticism on the relationship between T1 positive self-presentation on SNSs and T2 online upward social comparison was examined using simple slope analysis, with the results illustrated in Fig. 3. The findings demonstrated that for emerging adults who had high levels of T2 neuroticism, T1 positive self-presentation on SNSs significantly and positively predicted T2 online upward social comparison (*simple slope* = 0.036, *p* < 0.05). Conversely, for those who had low levels of T2 neuroticism, the predictive effect was non-significant (*simple slope* = 0.008, *p* = 0.371). These results indicate that positive self-presentation on SNSs was associated with stronger online upward social comparison among emerging adults high in neuroticism than among those low in neuroticism.

**Fig. 4:**
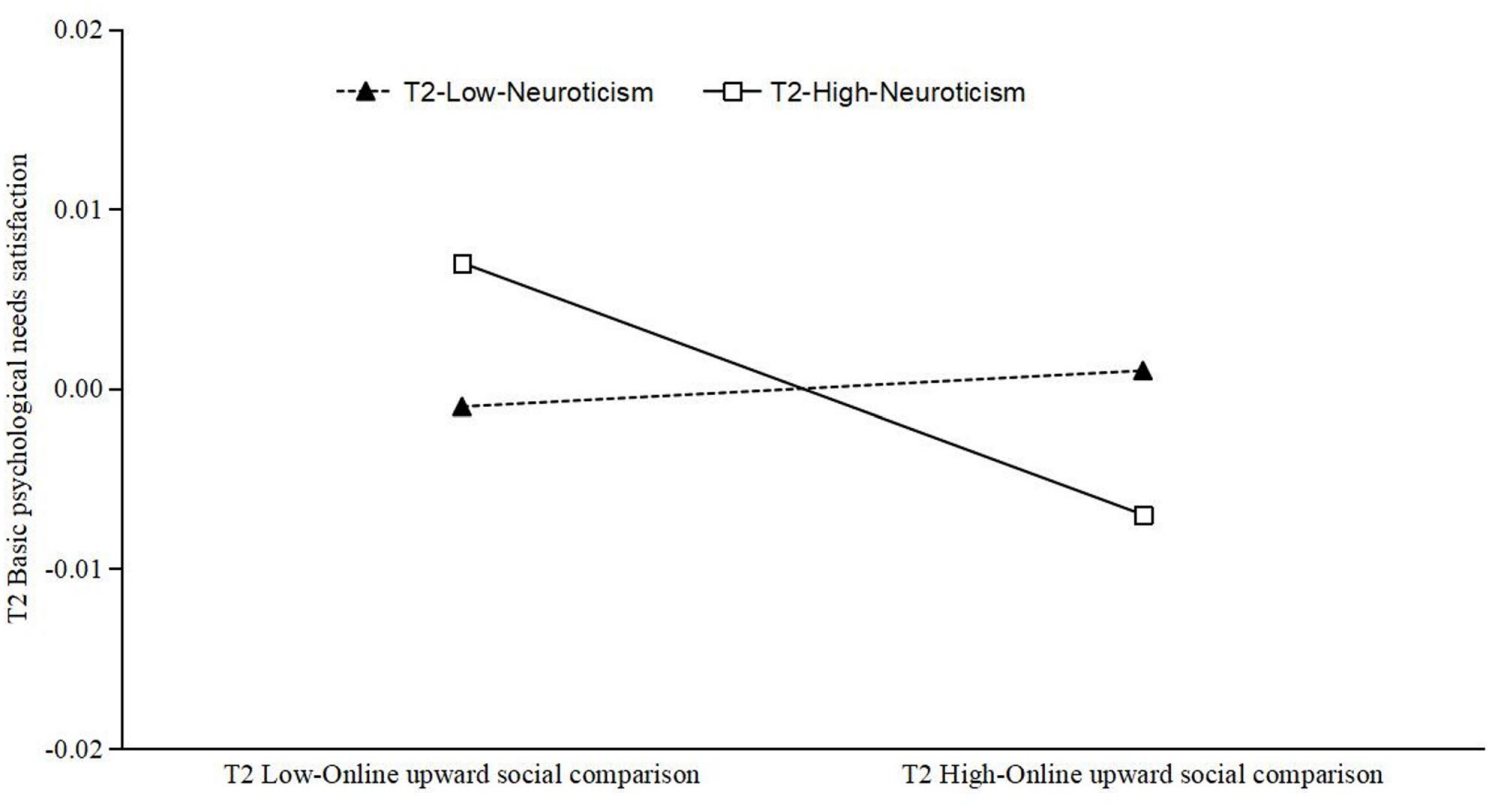
Simple slope analysis

The moderating effect of T2 neuroticism on the relationship between T2 online upward social comparison and T2 BPNS was examined using simple slope analysis, with the results illustrated in Fig. 4. For emerging adults who had high levels of T2 neuroticism, T2 online upward social comparison exerted a significant negative influence on T2 BPNS (*simple slope* = − 0.007, *p* < 0.05). Conversely, for emerging adults who had low levels of T2 neuroticism, T2 online upward social comparison did not contribute significantly to the prediction of T2 BPNS (s*imple slope* = 0.001, *p* = 0.471). These findings indicate that the negative association between online upward social comparison and BPNS was stronger among emerging adults high in neuroticism than among those low in neuroticism.

## Discussion

The ecological techno-subsystem theory posits that environmental factors, including digitally mediated contexts, play a significant role in shaping individuals’ psychological development [5]. In the digital age, the pervasive utilization of SNSs has increased experiences of FoMO among emerging adults [1]. As an important aspect of the online environment, self-presentation on SNSs and its impact on FoMO, as well as its underlying mechanisms, remain unclear. The present study employs a one-year, three-wave longitudinal design to explore the mechanisms through which self-presentation on SNSs influences FoMO. The objective of the present study is to undertake a comprehensive examination of the roles of individual cognitive factors (e.g., online social comparison), motivational factors (BPNS), and personality traits (neuroticism). Building on social comparison theory, self-determination theory, and the cognitive-affective system theory of personality, the study attempts to construct a model of individual-context interaction that explains how self-presentation on SNSs affects FoMO. The findings indicate that positive and authentic self-presentation on SNSs exert differential effects on FoMO through distinct pathways. Specifically, positive self-presentation on SNSs shows dual effects on FoMO: it reduces FoMO through the mediating role of BPNS, whereas it increases FoMO through a serial mediation pathway involving online upward social comparison and BPNS, with this indirect effect being particularly pronounced among individuals with high levels of neuroticism. In contrast, authentic self-presentation on SNSs reduces FoMO only through BPNS. Overall, online upward social comparison and higher levels of neuroticism increase the risk of experiencing FoMO, whereas BPNS functions as a protective factor against it.

### The longitudinal mediating role of online social comparison and basic psychological needs satisfaction between positive self-presentation on social networking sites and fear of missing out

The present study finds that T1 positive self-presentation on SNSs positively influences T3 FoMO through T2 online upward social comparison, partially validating Hypothesis 1a. The study results partially support the contrast effect of social comparison theory. The contrast effect posits that when individuals encounter social comparison information, their self-evaluation deviates from the comparison target, meaning that self-evaluation decreases during upward social comparison and increases during downward comparison [33, 34]. Individuals engage in upward social comparison based on the self-image presented on SNSs and the positive feedback they receive, as well as by comparing their attention to that given to other users’ profiles and status updates on SNSs [83]. It has been demonstrated that when emerging adults develop a tendency for online upward social comparison, they exhibit greater attention and sensitivity to the information of others. Frequent comparisons have been shown to engender feelings of stress and inferiority in emerging adults, which can result in increased social isolation. When emerging adults lack recognition for their abilities and self-worth, and struggle to form essential interpersonal connections, they may adopt passive coping strategies. This can lead to a persistent FoMO.

The present study also finds that T1 positive self-presentation on SNSs indirectly reduces T3 FoMO via T2 BPNS, thus providing support for Hypothesis 2a. This result is consistent with uses and gratifications theory [13], which suggests that positive self-presentation on SNSs fulfills basic psychological needs. It also aligns with self-determination theory [41], which posits that satisfying these needs enhances psychological well-being. Together, these findings point to a potential pathway where positive self-presentation mitigates FoMO. In other words, BPNS functions as a protective factor, mitigating the influence of positive self-presentation on FoMO. Collectively, these findings indicate that positive self-presentation on SNSs can partially alleviate FoMO by enhancing emerging adults’ satisfaction of basic psychological needs.

Moreover, the present study finds that T2 online upward social comparison and BPNS serially mediated the longitudinal association between T1 positive self-presentation on SNSs and T3 FoMO. This result provides partial support for Hypothesis 3a. The study suggests that the positive self-presentation of emerging adults on SNSs fosters online upward social comparison behavior, which in turn undermines BPNS and consequently leads to increased FoMO. This finding aligns with prior research, as evidenced by studies conducted by Boissicat et al. and Aldrup et al. [55, 58]. Specifically, the phenomenon of upward social comparison has been demonstrated to precipitate sentiments of inadequacy, thereby impeding BPNS for competence and autonomy. The present study further clarifies how positive self-presentation on SNSs influences FoMO. Online upward social comparison and BPNS are key mediators in this relationship. When individuals post positive or embellished content on SNSs, they often receive feedback such as likes or comments. This feedback can increase their tendency to engage in online upward social comparison [36]. The contrast effect of online upward social comparison suggests that people who frequently compare themselves to seemingly superior others on social media tend to experience lower self-esteem [30, 31]. This, in turn, leads to a reduction in BPNS, such as relationships and competence. Consequently, this leads to heightened FoMO [84]. Taken together, these findings indicate that online upward social comparison constitutes a critical risk factor in the relationship between positive self-presentation on SNSs and FoMO, increasing the likelihood of experiencing heightened FoMO.

### The longitudinal mediating role of online social comparison and basic psychological needs satisfaction between authentic self-presentation on social networking sites and fear of missing out

The present study shows that T1 authentic self-presentation on SNSs has an indirect negative effect on T3 FoMO via T2 BPNS. This supports Hypothesis 2b and emphasizes BPNS as a protective factor against FoMO in emerging adults. According to the uses and gratifications theory, individuals use SNSs primarily to satisfy specific psychological needs [13]. Individuals who engage in authentic self-presentation on SNSs can freely choose the content they share and their self-presentation style, thereby fulfilling their autonomy needs. At the same time, authentic self-presentation on SNSs can elicit more praise from friends, fulfilling their need for relationships [28]. This finding is also consistent with previous studies [85, 86]. Therefore, when needs are met, individuals can effectively self-regulate, thereby reducing the risk of FoMO.

Unlike positive self-presentation on SNSs, the present study shows that authentic self-presentation does not influence FoMO via online upward social comparison, and thus Hypothesis 1b is not supported. This finding is consistent with prior research indicating that embellishes or idealizes self-presentation, which characterizes positive self-presentation on SNSs, is more likely to elicit online upward social comparison, thereby intensifying FoMO [36, 37]. In addition, authentic self-presentation does not exert an effect on FoMO via the serial mediating role of online social comparison and BPNS. Thus, Hypothesis 3b is not supported. According to the criteria for serial mediation proposed by Hayes [87], a serial mediation pathway requires all constituent paths to be statistically significant. In the present study, the path from authentic self-presentation on SNSs to online social comparison is not significant. This serial mediation effect is not supported under both theoretical and statistical standards. Thus, in the serial mediation model, authentic self-presentation on SNSs can reduce FoMO only through the enhancement of BPNS, highlighting the protective role of BPNS. These findings suggest that authentic self-presentation has adaptive psychological value for FoMO. By allowing individuals to express their genuine thoughts, emotions, and life experiences [12], authentic self-presentation often elicits positive feedback from others [28], satisfying their needs for relatedness and competence, which in turn generates positive emotions and helps alleviate FoMO.

### The moderating role of neuroticism

The present study reveals that neuroticism significantly moderated the association between positive self-presentation on SNSs and online upward social comparison within the moderated mediation model. Specifically, positive self-presentation on SNSs is positively associated with online upward social comparison among emerging adults high in neuroticism, whereas this association is not significant among those low in neuroticism. This finding suggests that individuals with higher levels of neuroticism may be more sensitive to the implications of their self-presentation behaviors on SNSs, which may subsequently intensify their engagement in upward social comparison and further exacerbate FoMO. These findings provide partial support for Hypothesis 4a, indicating that neuroticism functions as a vulnerability factor that amplifies the risk-enhancing pathway from positive self-presentation to FoMO.

Neuroticism is characterized by emotional instability and a tendency toward negative affect [61]. This trait exerts a significant influence on the motivations for using SNSs. Prior research has shown that individuals high in neuroticism are more likely to present an idealized self [63]. Furthermore, they are more prone to engaging in online upward social comparisons [68]. One possible explanation is that individuals high in neuroticism tend to be more sensitive to their self-image and external evaluations, making them more vulnerable to emotional fluctuations triggered by social feedback. In the context of SNSs, these individuals engage more frequently in positive self-presentation on SNSs to gain social approval and positive feedback, thereby alleviating inner insecurity and anxiety [88]. Moreover, research suggests that individuals with high levels of neuroticism are more likely to experience envy when exposed to content depicting others in favorable situations [70]. Such emotional responses may motivate emerging adults to construct more idealized self-images and engage more frequently in online upward social comparisons. Furthermore, individuals with high neuroticism tend to exhibit stronger social competitiveness [69], which further amplifies their tendency toward online upward social comparison.

The present study also finds that neuroticism moderates the association between online upward social comparison and BPNS. The results indicate that online upward social comparison significantly and negatively predicted BPNS among emerging adults with high levels of neuroticism, whereas this association is not significant among those with low levels of neuroticism, thus providing partial support for Hypothesis 4b. Previous research has similarly indicated that social comparison tendencies are strongly influenced by neuroticism, with individuals high neuroticism tend to show stronger social comparison tendencies and greater susceptibility to jealousy [86]. Individuals with higher neuroticism often possess more negative and unstable self-concepts, and tend to rely more heavily on unfavorable social comparison information for self-evaluation, making them more vulnerable to the adverse consequences of negative social comparisons [89]. Consistent with the contrast effect in social comparison theory, individuals tend to experience more negative emotions when similar others appear better off, which may then lead to need deficiency.

These dynamics may collectively contribute to the development of FoMO, which likely arises from the complex interplay among multiple psychological processes. In addition, the present study finds a significant negative association between neuroticism and BPNS, consistent with previous research [71]. Future studies may further explore the mechanisms underlying the relationships among these variables.

It is important to note that positive and authentic self-presentation on SNSs differ in their directional effects and underlying psychological mechanisms in shaping FoMO. The present study finds that although both forms of self-presentation on SNSs can reduce FoMO by enhancing BPNS, their effects are characterized more by differences than similarities. Specifically, positive self-presentation on SNSs increases FoMO by promoting online upward social comparison, which in turn reduces BPNS and ultimately intensifies FoMO among emerging adults. In contrast, authentic self-presentation on SNSs neither significantly predicts online upward social comparison nor influences FoMO through the proposed serial mediation pathway. This discrepancy may stem from the generally positive bias in self-presentation on SNSs, where individuals tend to highlight favorable traits and enjoyable experiences, such as posting attractive selfies or sharing positive life events [90]. Furthermore, neuroticism plays a differential moderating role: it amplifies the serial indirect effect of positive self-presentation on SNSs on FoMO via online upward social comparison and BPNS, but does not moderate the effects of authentic self-presentation.

Based on these findings, interventions aim at reducing FoMO among emerging adults should target both risk factors and protective psychological processes. First, educational programs should increase individuals’ awareness of how positive self-presentation and upward social comparison on SNSs contribute to FoMO. Emerging adults are encouraged to adopt more authentic self-presentation strategies on social media. Given that exposure to highly curated online content can intensify upward social comparison, fostering critical reflection on such content and reducing excessive reliance on social feedback may help attenuate the risk pathway from self-presentation to FoMO. Second, interventions should focus on enhancing BPNS, which consistently emerges as a protective factor against FoMO. Creating autonomy-supportive, competence-enhancing, and socially supportive environments may strengthen emerging adults’ self-regulation. These environments can be established both online and offline and may reduce their vulnerability to FoMO. Moreover, individuals with high levels of neuroticism may be particularly susceptible to the negative effects of positive self-presentation on SNSs and online upward social comparison. For this group, interventions emphasizing emotional regulation and adaptive coping strategies may be especially beneficial. Collectively, these findings suggest that FoMO interventions should integrate self-presentation strategies on SNSs, social comparison awareness, basic psychological needs support, and personality sensitive strategies. This would help emerging adults adapt more effectively to digital social environments.

### Strengths, limitations and further direction

This study makes a novel theoretical contribution by highlighting the complex role of self-presentation on SNSs in shaping FoMO. Using a one-year, three-wave longitudinal design, the findings demonstrate that cognitive factors (online upward social comparison), motivational factors (BPNS), and personality traits (neuroticism) jointly play significant roles in the impact of self-presentation on SNSs on FoMO among emerging adults. With respect to FoMO, positive self-presentation on SNSs does not function solely as either a protective or a risk factor. Rather, it exerts dual and opposing effects through distinct pathways: a risk pathway via online upward social comparison, either independently or in combination with reduced BPNS, and a protective pathway via BPNS. These pathways operate in opposite directions and partially offset each other, highlighting how FoMO is simultaneously shaped by comparison-induced risks and need-based resources in digital contexts. Furthermore, individual differences, such as neuroticism, moderate the relative strength of these pathways, underscoring the person-dependent nature of self-presentation effects. This study develops a dual-pathway, double-edged effect model, moving beyond simplistic dichotomies that frame self-presentation as purely beneficial or harmful. It provides a more nuanced framework for understanding the psychological mechanisms of FoMO in modern digital environments.

Despite these contributions, several limitations should be acknowledged. First, although longitudinal designs provide valuable insights into temporal associations among variables, they remain non-experimental in nature, and thus preclude definitive causal inferences. Second, the exclusive reliance on self-report questionnaires may have introduced common method bias and other unmeasured confounding factors. To address these limitations, future research should employ experimental approaches (e.g., priming paradigms) to manipulate self-presentation on SNSs and online social comparison, thereby allowing for more rigorous tests of causal relationships. Third, the duration and frequency of the assessment period could be further improved. Although the present study used a three-wave longitudinal design with six-month intervals, future research could employ longer and more frequent assessments to better capture the dynamic processes linking self-presentation, online social comparison, BPNS, and FoMO. Fourth, the gender distribution in the present sample was relatively imbalanced, largely reflecting the inherent gender composition of students across the surveyed universities and academic majors. Although gender was statistically controlled in the analyses, future research should further examine the robustness of the present findings using samples with a more balanced gender distribution. In addition, subsequent studies could explore potential gender differences in the relationships among the key variables investigated in this study. Finally, although the present findings demonstrate that positive self-presentation on SNSs influenced FoMO through a serial mediation pathway involving online upward social comparison and BPNS, the observed effect sizes were relatively small. Future research should replicate and validate these relationships in larger and more diverse samples to establish their robustness and generalizability.

## Conclusions

This study elucidates the mechanisms through which self-presentation on SNSs influences FoMO among emerging adults over a one-year period, highlighting a serial mediation process involving online upward social comparison and BPNS, as well as the moderating role of neuroticism. The results demonstrate that positive and authentic self-presentation on SNSs exert differential effects on FoMO through distinct pathways. Specifically, positive self-presentation on SNSs exert dual effects on FoMO. On one hand, they can alleviate FoMO by enhancing BPNS. On the other hand, they may intensify FoMO by promoting online upward social comparison, which subsequently undermines BPNS. Moreover, neuroticism moderates these effects: individuals high in neuroticism are more likely to engage in online upward social comparisons as their positive self-presentation increases, which in turn decreases BPNS and intensified FoMO. In contrast, authentic self-presentation reduces FoMO only through increases BPNS.

Taken together, these findings indicate that positive self-presentation on SNSs simultaneously activate protective and risk mechanisms in shaping FoMO. BPNS functions as a critical protective factor, whereas online upward social comparison operates as a risk mechanism, either independently or through its detrimental effect on BPNS. This risk process may be amplified under certain individual characteristics, such as higher levels of neuroticism. This study advances a nuanced understanding of the psychological outcomes of self-presentation on SNSs and lays a solid theoretical foundation for educational and psychological interventions aimed at mitigating FoMO in emerging adults.

## Data Availability

The datasets used and/or analysed during the current study are available from the corresponding author on reasonable request.

## Abbreviations

SNSs: Social networking sites
FoMO: Fear of missing out
BPNS: Basic psychological needs satisfaction
SNSs-P: Positive self-presentation on SNSs
SNSs-A: Authentic self-presentation on SNSs
OSC-D: Online downward social comparison
OSC-U: Online upward social comparison
